# Developing a Vial-Scale Methodology for the Measurement of Nucleation Kinetics Using Evaporative Crystallization: A Case Study with Sodium Chloride

**DOI:** 10.1021/acs.cgd.4c01722

**Published:** 2025-04-04

**Authors:** Michele Chen, Leif-Thore Deck, Luca Bosetti, Marco Mazzotti

**Affiliations:** Institute of Energy and Process Engineering, ETH Zürich, 8092 Zürich, Switzerland

## Abstract

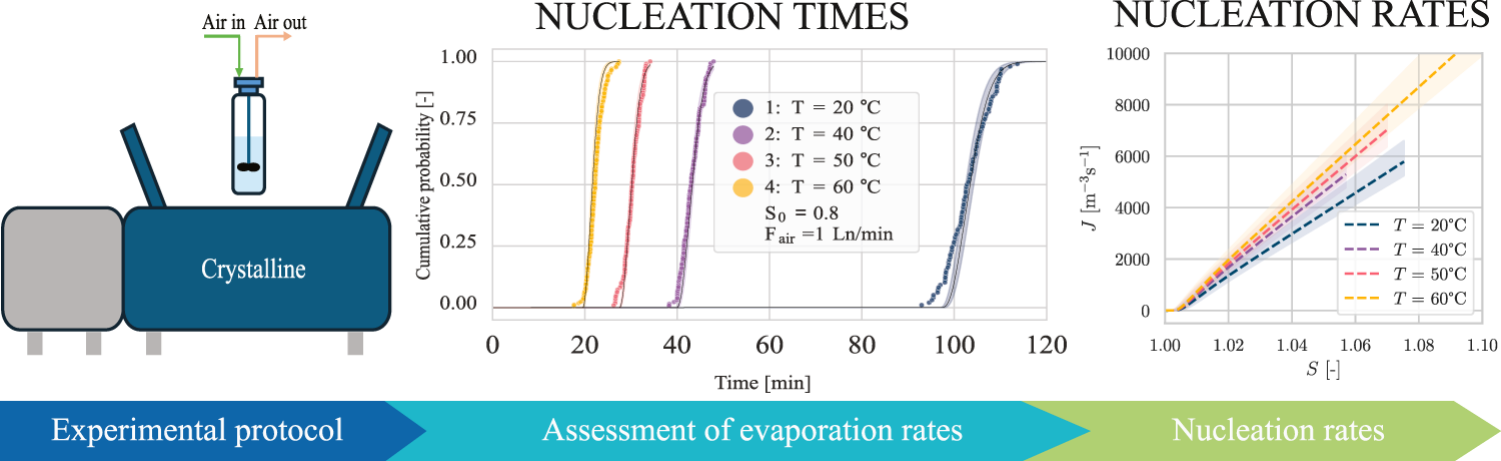

Understanding nucleation kinetics is vital for designing crystallization processes, yet traditional measurement methods based on cooling are unsuitable for compounds with temperature-independent solubility. This study introduces an experimental procedure to measure the nucleation kinetics based on evaporative crystallization and applies it to sodium chloride (NaCl) in water. By systematically varying the experimental conditions such as temperature and evaporation gas flow rate, we obtained a comprehensive data set for the nucleation of NaCl crystals that allowed estimating kinetic parameters using a rate expression derived from classical nucleation theory (CNT). This work demonstrates the robustness of evaporation as a method for measuring the nucleation kinetics that is applicable regardless of how the solubility of a compound depends on the temperature.

## Abstract 2

This study introduces a vial-scale evaporative crystallization method to measure nucleation kinetics applied to aqueous sodium chloride (NaCl). By integrating experimental data and classical nucleation theory, it highlights the influence of evaporation rates on supersaturation and metastable zone width. The method demonstrates reproducibility and scalability, offering a robust framework for crystallization studies.

## 1 Introduction

Primary nucleation refers to the initial formation of crystalline particles from a supersaturated solution without the involvement of existing crystals. It plays a fundamental role in crystallization processes, where its rate determines the number and solid form of the resulting crystals. However, nucleation events are rare, occur on microscopic scales, and happen within very short time frames, making direct observation and tracking of individual events challenging.^1^ The single-nucleus hypothesis proposes that once a stable nucleus forms within a small volume, it initiates a cascade of secondary nucleation events and crystal growth that makes the new phase macroscopically visible, with stochasticity associated with the primary nucleation event.^2−4^ To capture the overall behavior and variability, meaningful studies of nucleation require large experimental data sets. Nucleation times can vary significantly even under identical experimental conditions: nucleation is an activated process where a stable nucleus is formed only by overcoming an energy barrier through energy fluctuations, which leads to nucleation times being distributed quantities. As a result, nucleation times are best described using probabilistic approaches.^2,4−8^ The stochasticity of primary nucleation is particularly evident at small scale (e.g., in microdroplets or vials)^5,9,10^ and has been observed for a wide variety of crystallizing systems, including organic, inorganic, and biological molecules.^6,11,12^

Sodium chloride (NaCl) is a well-established model compound for crystallization studies due to its ubiquity and well-characterized properties.^13−18^ Notably, NaCl has a solubility that is relatively insensitive to temperature changes, making solvent evaporation a more effective method for inducing supersaturation than cooling.^19,20^ In industrial settings, evaporative crystallization is the primary method for producing salt from brines,^21,22^ highlighting the need for a deeper understanding of nucleation kinetics in this system.

Crystallization of NaCl has been studied under a range of different conditions: microdroplets,^23,24^ acoustically levitated droplets,^25^ cooling crystallization,^26^ antisolvent crystallization,^27,28^ and electrodynamically trapped aerosol droplets.^29^ However, these studies often involve conditions that are difficult to translate to practical, larger-scale systems. While the microdroplet scale offers high throughput, it comes at the cost of high complexity because the nucleation models must account for the flow within the droplet that is driven by the volume change in time.^30−32^ Multiple studies^23,24,32,33^ have shown that evaporating droplets exhibit intricate flow patterns that require advanced modeling to describe the volume evolution over time. Despite the widespread application of NaCl evaporative crystallization,^34^ experimental data on NaCl nucleation kinetics at the vial scale (1–10 mL) remain limited.^26^

Evaporative crystallization, although more labor-intensive than cooling crystallization due to discontinuous measurements from solvent depletion, enables the study of nucleation kinetics driven purely by solvent removal at constant temperature and the separate assessment of the evaporation rate determined by heating or by the flow rate of the evaporation gas.

In this study, we introduce a workflow for measuring nucleation rates by evaporative crystallization at the vial scale and apply it to sodium chloride (NaCl). The approach includes both the experimental procedure utilizing established crystallization laboratory equipment and a data analysis method for nucleation data sets obtained under different conditions. We estimate nucleation parameters using a rate expression derived from classical nucleation, quantify their uncertainties, and assess the impact of experimental variability on the nucleation times using experiments that explore the effects of temperature, evaporation rate, and initial saturation ratio. Our findings demonstrate the reproducibility and reliability of the proposed method.

## 2 Materials and Methods

### 2.1 Experimental Setup

**Figure 1:**
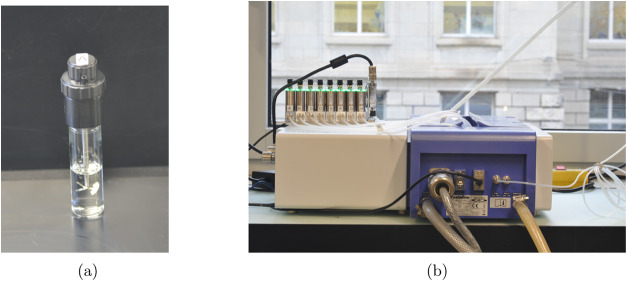
(a) Vial with an evaporation cap and a short-blade impeller. (b) Crystalline device with the eight mass flow controllers (MFCs) and silicone tubing to connect the eight vials.

Nucleation studies are commonly conducted using parallelized crystallization systems to enable higher throughput and automation. In this work, we utilized the Crystalline system (Technobis Crystallization Systems), a commercial device featuring eight parallel and independent reactors with temperature and agitation control, widely used for nucleation and screening studies.^26,34−43^ Each reactor consists of an 8 mL glass vial (catalog number 1050-4463, Fisher Scientific) equipped with dedicated stirrers and caps tailored for specific applications. Nucleation detection was achieved by measuring transmissivity using a laser sensor, an established and highly sensitive method in nucleation studies.^7,8^ The Crystalline system is equipped with eight evaporation gas supply channels, whose flow rates are, however, not controlled: to ensure a repeatable and constant supply, we provided dry air as the evaporation gas through eight separate thermal mass flow controllers (Flexi-Flow A2K0, Bronkhorst Co., The Netherlands), each providing up to 2 normal liters per minute (Ln/min), which means an amount of gas equivalent to 2 L/min at normal conditions, i.e., 0 °C and 1 bar. We used a fill volume for the vials of 4 mL to prevent splashing of the solution due to the high evaporation gas flow rates (see Figure 1).

The vials were stirred at a rate of 1250 rpm using the proprietary short-blade impellers to ensure sufficient mixing and prevent crystals nucleated at the air–solution interface from remaining there. The use of magnetic stir bars (8 × 3 mm, 001.808.3, Fisherbrand) proved insufficient to ensure suspension of the crystal mass compared to overhead stirrers.

Vial caps equipped with stirrers and mass flow controllers (MFCs) were labeled A–H and consistently used in the same positions on the Crystalline system for all experiments. New vials were utilized for each experiment to prevent any cross contamination.

### 2.2 Experimental Procedure

#### 2.2.1 Materials

Sodium chloride (puriss. p.a., ≥99.5% (AT), Sigma-Aldrich) was used without further purification. Ultrapure water (resistivity >18 MΩ cm), produced in-house using a Milli-Q Advantage A10 system (Millipore), was utilized for solution preparation and final rinse of reusable glassware.

#### 2.2.2 Preparation of Solutions

Stock solutions for the experimental runs were prepared by dissolving specified amounts of sodium chloride (NaCl) in 400 g of ultrapure water under stirred conditions at ambient temperature (Table 1), targeting a specified initial saturation ratio. The saturation ratio is defined as *S* = *C*/*C*_NaCl_^*^, where *C*_NaCl_^*^ is the solubility of NaCl in water obtained from measurements by Flannigan et al.^26^ The expression for the solubility obtained by regression is *C*_NaCl_^*^ = 0.3541 + 0.0002*T* [kg_NaCl_kg_H_2_O_], where the temperature is given in °C. After dissolution, the solutions were filtered using a 0.22 μm hydrophilic PTFE syringe filter (BGB) to remove any undissolved solids and impurities. The filtered solutions were then dispensed into glass vials using a 100–1000 μL pipet (Finnpipette F2, Thermo Fisher), following an adaptation of an experimental protocol already in use for nucleation measurements.^8,44^

**Table 1:** Composition of the Stock Solutions Used for the Preparation of Aqueous NaCl Solutions with Initial Saturation Ratios of 0.8 and 0.9

*m*_NaCl_ [g]	*m*_H_2_O_ [g]	*S*_0_
112	400	0.8
126	400	0.9

#### 2.2.3 Assessment of Evaporation in the Vials

To verify the assumption of a constant evaporation rate and assess measurement reproducibility, we characterized the evaporation by tracking the mass loss in each vial containing NaCl solutions using a high-precision balance (AX205, Mettler Toledo). These measurements were performed at a fixed temperature and gas flow rate. The evaporation rate, *ṁ*_*v*_, was calculated from the gravimetric measurements both throughout the process and within shorter time intervals. Our measurements at 20 and 40 °C confirmed that the evaporation rate remained constant throughout the experiments (see S1 in the Supporting Information for details).

The variability in the evaporation rate and initial solution mass between experiments was accounted for during the fitting of the nucleation rate parameters, as discussed in Section 2.5.

#### 2.2.4 Measurement of Nucleation Times

Each experiment began with a 10 min equilibration step at the set-point temperature. Representative solution temperatures for the vials were measured for each condition using external temperature probes (K-Type 607013, Telstar) and a temperature logger (TC-08, Pico Technology). The experiments were carried out at a fixed stirring rate of 1250 rpm.

Nucleation events were identified based on the time at which a marked and sustained decrease in the transmissivity signal occurred, as detected by the Crystalline device. In doing so, we assumed that there is no relevant delay between the initial nucleation event and the detection, which is accurate if crystals grow rapidly.^45^ A custom tool developed in Python was used to analyze the transmissivity data.

##### Temperature Variation

To investigate the effect of temperature, we conducted evaporation experiments on solutions with an initial saturation ratio of *S*_0_ = 0.8 at temperatures of 20, 40, 50, and 60 °C. The flow of air from the MFCs was 1 Ln/min in these experiments.

##### Air Flow Rate Variation

Three air flow rates were applied, namely, 1, 1.5, and 2 Ln/min, through the mass flow controllers at a fixed solution temperature of 40 °C and an initial saturation ratio of 0.8.

##### Initial Saturation Ratio Variation

We performed additional experiments at an initial saturation ratio of 0.9, 20 °C, and an air flow rate of 1 Ln/min. This condition serves as a verification of the reproducibility of the experimental measurement.

A summary of the experimental series and the number of independent measurements (corresponding to the number of vials) conducted under each condition is provided in Table 2.

**Table 2:**
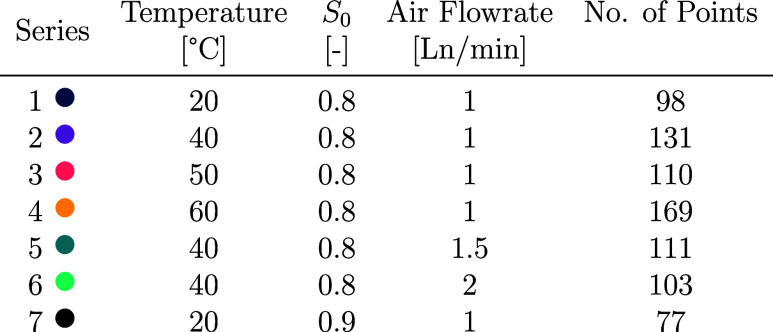
Summary of Experimental Conditions Explored, Varying Temperatures *T*, Air Flow Rates *F*_air_, and Initial Saturation Ratios (*S*_0_)^a^

a For all conditions, the vial fill volume is 4 mL and the stirring rate is 1250 rpm. The same color-coding is maintained throughout the rest of this work.

### 2.3 Modeling Evaporation

To model the nucleation of NaCl in the Crystalline setup, two components are needed: (i) an evaporation model to describe the evolution of the solution’s volume, concentration, and supersaturation over time and (ii) a nucleation model to describe the effect of the experimental conditions (temperature, supersaturation) on nucleation. We developed a mathematical model under the assumption of a constant evaporation rate. This simplification is justified by the controlled experimental conditions and the uniform gas flow supplied to each vial. Modeling evaporation allowed us to predict the supersaturation profile over time. In each experimental run, the key measurable quantities for each vial are the initial mass of NaCl in solution *m*_*s*,0_, the initial mass of water in solution *m*_*w*,0_, known from the preparation of the solutions, the time to nucleation *t*_nuc_, recorded by the transmissivity sensor of the Crystalline device, and the evaporation rate *ṁ*_*v*_, calculated from the total evaporated mass determined by gravimetry.

The evolution of the mass of water as a function of time is given by
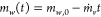
1The mass of the solute NaCl is constant, and its mass fraction is calculated as
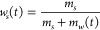
2The volume of the solution is obtained from the total mass and the density of the solution:
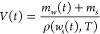
3where ρ(*w*_*s*_, *T*) is the composition- and temperature-dependent solution density, calculated using correlation ρ(*w*_*s*_, *T*) = 995.3 + 753.3 *w*_*s*_ – 8.442·10^–3^*T*^2^ provided by Carvalho et al.,^46^ where temperature is given in °C and ρ is in kg m^–3^.

Finally, we defined the saturation ratio as the ratio of the actual salt concentration to its equilibrium concentration, *c*_s_*(*T*):
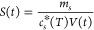
4This model calculates the time-dependent changes in solution composition, volume, and saturation ratio during evaporation. To compare experiments at different evaporation rates, it is worth introducing the dimensionless time, τ:
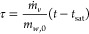
5where *t*_sat_ is the time at which the solution becomes saturated, defined using eq 4 by *S*(*t*_sat_) = 1. We reformulated the evaporation model in terms of the dimensionless time as follows:
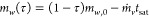
6
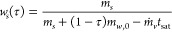
7
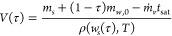
8
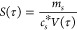
9

### 2.4 Modeling Nucleation

Since both the volume and the saturation ratio change during the evolution of the experiment, we modeled nucleation as a nonhomogeneous Poisson process.^6,8,24,47,48^ We assumed that the appearance of the first nucleus determines the outcome of the experiment (also called single-nucleus mechanism).^3^ Given a nucleation rate *J*, representing the number of nuclei formed per unit volume and per unit time by primary nucleation, the cumulative probability, *P*, for a sample to nucleate between the beginning of the experiment and time *t*_nuc_ is given by
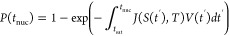
10where the mass of solvent changes over time, so that volume and saturation ratio are time-dependent as well. Note that *t*_sat_ is the time when the solution becomes saturated (see eq 5), which depends deterministically on the initial salt concentration and the evolution of the evaporation. A similar approach was followed by Goh et al.^6^ for lysozyme and used by Cedeno et al.^24^ for NaCl in microdroplets.

To model the nucleation rate’s dependence on temperature and initial solute mass, we employed the classical nucleation theory.^49^ The use of the CNT to describe the nucleation of NaCl is appropriate, as the literature consensus attributes a classical behavior to nucleation occurring in the low-supersaturation region, which is where experimental results fall in our experimental campaign,^18^ while a two-step mechanism has been proposed for the high-supersaturation region.

We therefore used as nucleation rate expression:^49^
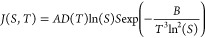
11where *A* is a prefactor, *D*(*w*_*s*_, *T*) is the diffusivity of NaCl in solution, and the exponential term characterizes the dimensionless work required to form a stable nucleus. The unit of *A* is m^–5^, while *B* is in K^–3^, and both parameters are considered to be temperature-independent. We expressed the diffusivity using the Stokes–Einstein equation:
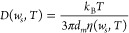
12where *k*_B_ is the Boltzmann constant and η(*w*_*s*_, *T*) is the kinematic viscosity of the solution, which depends on the mass fraction of NaCl and on the temperature (*T*). The molecular diameter *d*_*m*_ is calculated as , where *v*_*m*_ is the molecular volume of NaCl equal to *v*_*m*_ = 1/ρ_*s*_. The solid density ρ_*s*_ in molecular terms was assumed to be equal to 2.27·10^28^ m^–3^. The kinematic viscosity η(*w*_*s*_, *T*) was derived from the dynamic viscosity and density of the solution as η(*w*_*s*_, *T*) = μ(*w*_*s*_, *T*) /ρ(*w*_*s*_, *T*), both temperature- and concentration-dependent. Correlation μ(*w*_*s*_, *T*) = 2.367 + 2.205 *w*_*s*_ – 0.491ln(*T*) was taken from Carvalho et al.^46^ and provided the dynamic viscosity [mPa s] from the mass fraction of salt and temperature [°C]. It is worth mentioning that the diffusivity changes by about 11% due to temperature in the relevant range and by 1% due to the mass fraction of NaCl, enough to be considered in the model.

The nucleation probability can be recast in terms of dimensionless time τ, defined in eq 5 as
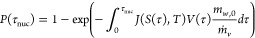
13where τ_nuc_ is the nucleation time *t*_nuc_ converted to the dimensionless form using eq 5.

### 2.5 Parameter Estimation

First, outliers were identified and excluded using the interquartile range (IQR) method. Within each series of data listed in Table 2, the IQR was calculated for the relevant quantities: the evaporation rate and initial mass of the solution. For each quantity, outliers were defined as values falling outside 1.5 times the IQR below the first quartile (Q1) or above the third quartile (Q3). The indices of these outliers were collected and removed from the data set. A total of 46 experimental points out of 845 measurements were excluded.

Initial guess values were obtained by using simulations of the nucleation process to produce distributions of nucleation times compatible with experimental measurements. By providing informed starting points, we improved the likelihood of the optimization routines converging to a global minimum.

We aimed to describe the entire experimental data set using the single expression for the nucleation rate given by eq 11, thus going beyond the more common approach whereby the nucleation rate parameters are estimated separately for each experimental condition.^50,51^

To construct the objective function, which compares the empirical CDFs with the calculated CDFs, we used the nucleation times obtained experimentally and converted them to the dimensionless form and the calculated dimensionless nucleation times. The probability values *P*_exp_ of empirical CDFs are defined as
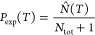
14where *N*_tot_ is the total number of experimental points and *N̂*(*T*) is the number of experiments nucleated in the interval [*T*^eq^, *T*].^44^ The values of the CDFs were calculated using the ECDF function implemented in the statsmodel package in Python.^52^ The calculated nucleation times were then obtained using the probability values of the empirical CDF and solving eq 13 to τ_nuc_.^44^

The optimization problem can be formulated using the sum of the squared errors relative to the geometric mean of the nucleation times as^53^
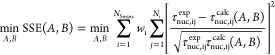
15where the weighted sum of squared errors (SSE) is the objective function to minimize. The objective function sums the relative errors over multiple experimental conditions *N*_Series_, as summarized in Table 2: we used series 1–4 to perform the parameter estimation. The contribution of each experimental condition was weighed using weight *w*_i_, defined as the inverse of the fraction of experiments for the experimental series with respect to the total number of experimental points for all conditions *w*_i_ = *N*/*N*_*i*_; thus, the estimation is not biased by sets with more points. Furthermore, nucleation parameters were transformed by using a logarithmic scale in the optimization routine to reach convergence more smoothly. Parameter estimation was performed using Python 3.9.13, utilizing the minimize function from the scipy.optimize package with the Nelder–Mead simplex algorithm,^54^ a derivative-free method suitable for the form of our objective function. The tolerance in the solver was set to 1 × 10^–30^. This approach is in line with previous works on the estimation of crystal nucleation kinetics.^8,44^

### 2.6 Confidence Intervals

Confidence intervals of the nucleation parameters estimated through the nonlinear optimization procedure were calculated using a nonparametric bootstrap resampling approach.^55^ This method was selected due to the complexity of the objective function, which precluded the reliable use of the variance–covariance matrix method. The original data set was resampled with replacement to generate 1000 bootstrap samples, each containing the same number of observations as the original data set. Resampling ensured that some data points were duplicated, while others were omitted in each bootstrap sample. Compared to parametric Monte Carlo methods, the bootstrap approach directly uses experimental data and is less sensitive to small-sample biases.^56,57^ For each bootstrap sample, the optimization procedure was repeated to re-estimate the model parameters, with each iteration providing a new set of parameter estimates, forming an empirical distribution of parameter values. The distributions of the bootstrapped parameter estimates were analyzed to determine the 95% confidence intervals.

### 2.7 Quantification of Variability

Variability in nucleation experiments can be attributed to different sources: the experimental error from the preparation of samples, the experimental procedure, the measurement device and the method of detection, and the stochasticity of nucleation itself.^44^ The importance of quantifying the experimental error lies in the ability to compare it with the stochasticity due to nucleation; *a priori*, it is not clear whether this stochasticity or experimental error is the dominant source of the measured variability in nucleation times.^4,58−60^ By identifying and excluding all relevant types of experimental errors, the remaining variability can be attributed to the stochasticity of nucleation.

**Figure 2:**
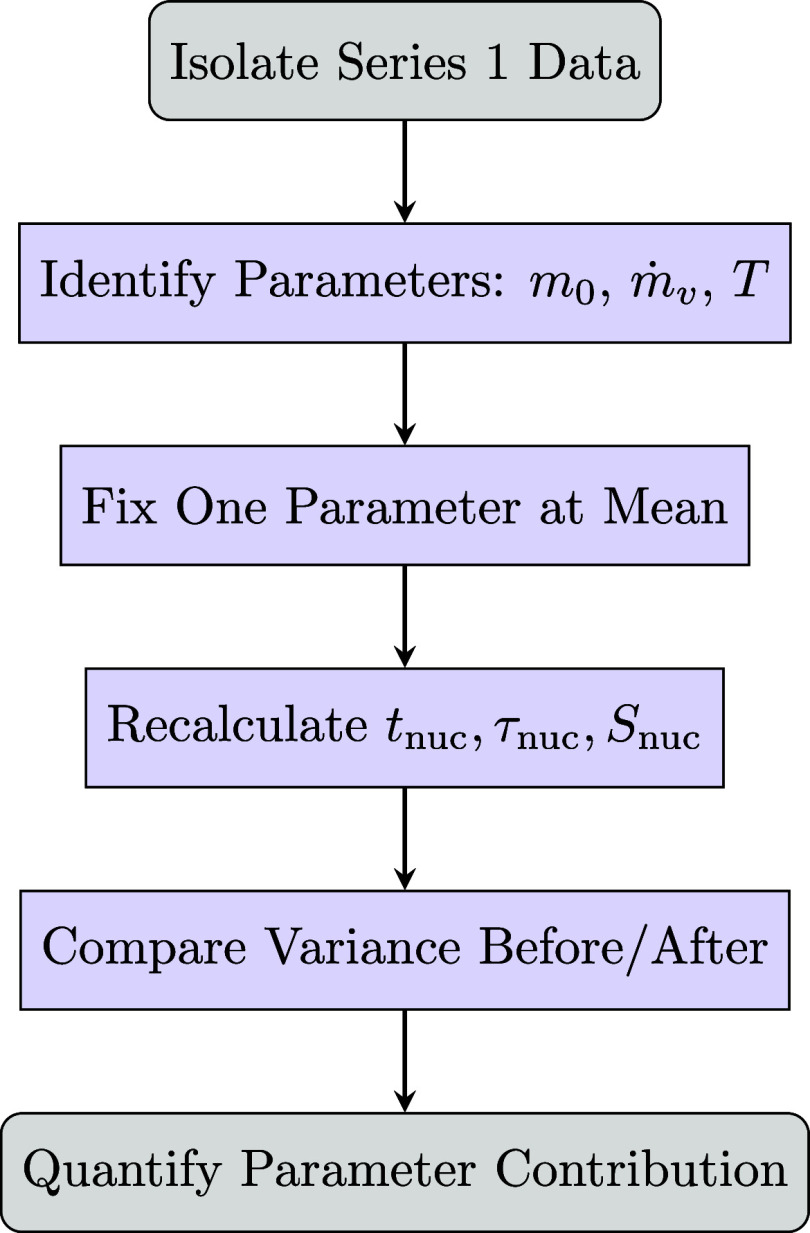
Compact flowchart illustrating the variance decomposition procedure. Each key parameter (*m*_0_, *ṁ*_*v*_, *T*) is systematically fixed, and variance is recomputed to assess its contribution to overall variability.

To assess how variability in key input parameters influences the distribution of nucleation times, we performed a variance decomposition analysis on experimentally measured nucleation times, dimensionless nucleation times, and supersaturation levels;^61^ this protocol is illustrated in Figure 2. First, we reduced the experimental data set to a specific operational condition (for this analysis, we chose series 1, with fixed initial concentration *S*_0_, air flow rate, and temperature), but the conclusions can be extended to the other conditions. We then systematically isolated each parameter, i.e., initial mass *m*_0_, evaporation rate *ṁ*_*v*_, and temperature *T*, by fixing it at its mean value and recalculating the nucleation metrics while allowing the other parameters to vary. By comparing the variance obtained with and without fixing a given parameter, we quantified that parameter’s contribution to the overall variability. Specifically, for the temperature, we investigated whether variations in individual vial temperatures, independent of their effect on the evaporation rate, significantly influenced the observed experimental variability.

We first determined nucleation times *t*_nuc_ for each experimental run by numerically solving the following expression: *S*_nuc_^exp^ – *S*(*t*) = 0 for *t*, where *S*(*t*) is given by eq 4. In this step, we set *S* to the vial-specific supersaturation values, thus accounting for the actual measured conditions. To isolate the influence of individual parameters, we fixed one parameter at its mean value while still allowing the others to vary according to their measured distributions. After determining *t*_nuc_ under these fixed-parameter scenarios, we recalculated the dimensionless nucleation times τ_nuc_ using eq 5 and the corresponding *t*_sat_ values to maintain consistent scaling. We also computed the supersaturation at nucleation *S*_nuc_ from eq 4 under each scenario. By performing these recalculations for every data point in the reduced data set, we obtained new distributions of *t*_nuc_, τ_nuc_, and *S*_nuc_, from which we analyzed how the variances changed when the variability of individual parameters was removed.

This procedure allowed for a direct comparison between the total experimental variance (reflecting all natural experimental fluctuations) and the reduced-variance scenarios where one parameter’s variability was removed. The difference between these variances serves as a measure of that parameter’s relative importance in controlling the observed range of nucleation metrics.

Finally, we also estimated the relative error associated with the gravimetric measurements and its propagation to the calculation of evaporation rates, volumes, and supersaturations and the error associated with the correlation for the solubility to find that it does not affect our ability to detect nucleation events (see S4.1 in the Supporting Information).

## 3 Results

### 3.1 Nucleation Time Measurements

**Figure 3:**
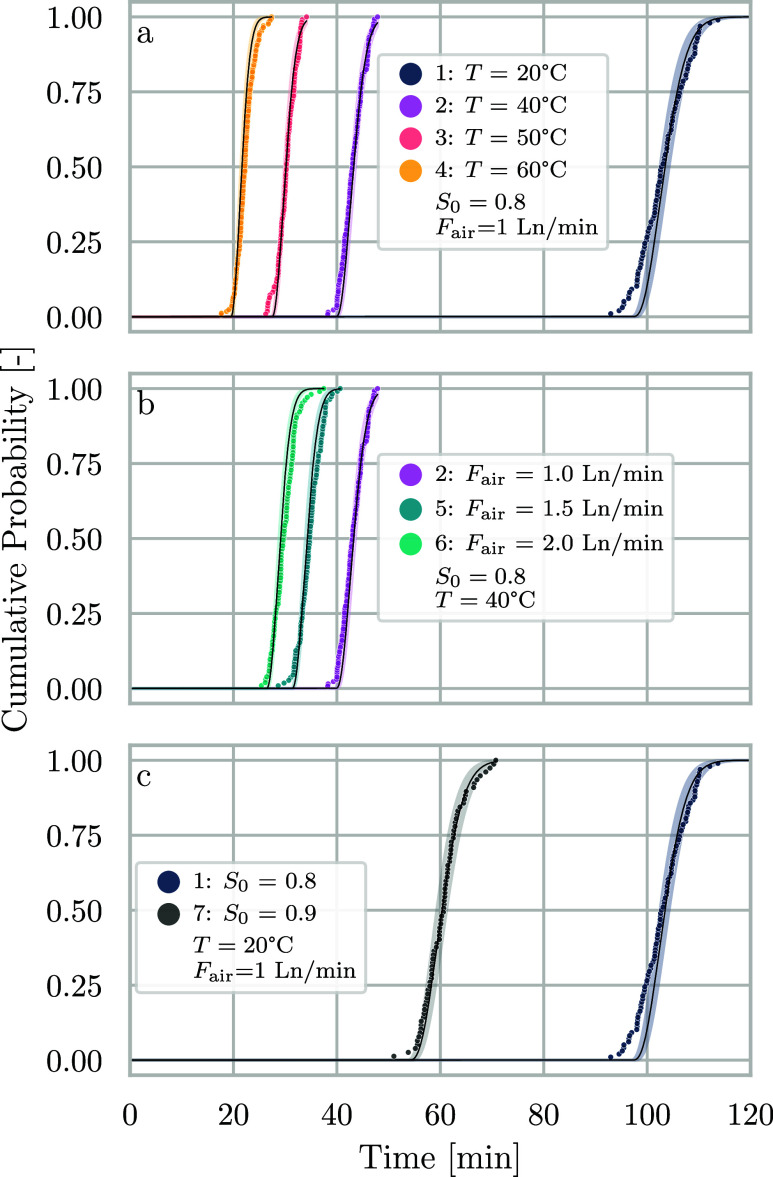
Cumulative probabilities of nucleation times for NaCl at the conditions reported in Table 2. The scatter points represent experimental data, solid lines are model curves using fitted parameters, and shaded areas represent their 95% confidence intervals. (a) Series 1–4 at different levels of temperature. (b) Series 2, 5, and 6 at different flow rates of air. (c) Series 1 and 7 at different initial saturation ratios. Gas flow rates are measured in normal liters per minute, i.e., 0 °C and 1 bar.

Next, we analyzed the results of the experimental campaign: we used the experimental procedure and the models introduced in the previous section to interpret the nucleation times measurements performed under the conditions reported in Table 2. The experimental results are shown in Figure 3 as cumulative distribution functions (CDFs) of nucleation times for the conditions summarized in Table 2.

Figure 3a reports the four experiments (series 1–4) performed at different temperatures, with a fixed flow rate of air of 1 Ln/min and an initial concentration *S*_0_ of 0.8. Temperature affects the rate of evaporation, as evaporation is determined by both the heat flux provided to the system and the rate at which vapor-phase water is removed from the vial. Overall, the set-point temperature affects both the nucleation kinetics and the evaporation rate of the solvent. The effect of the evaporation alone is illustrated in Figure 3b, where series 4, 5 and 6 were performed at varying flow rates of air, fixed temperature (*T* = 40 °C) and fixed initial concentration (*S*_0_ = 0.8). Finally, we show the comparison of series 1 and series 7 in Figure 3c, where the initial concentration is varied, while the flow rate of evaporation gas is kept at 1 Ln/min and the temperature is fixed at *T* = 20 °C.

### 3.2 Supersaturation at Nucleation

**Figure 4:**
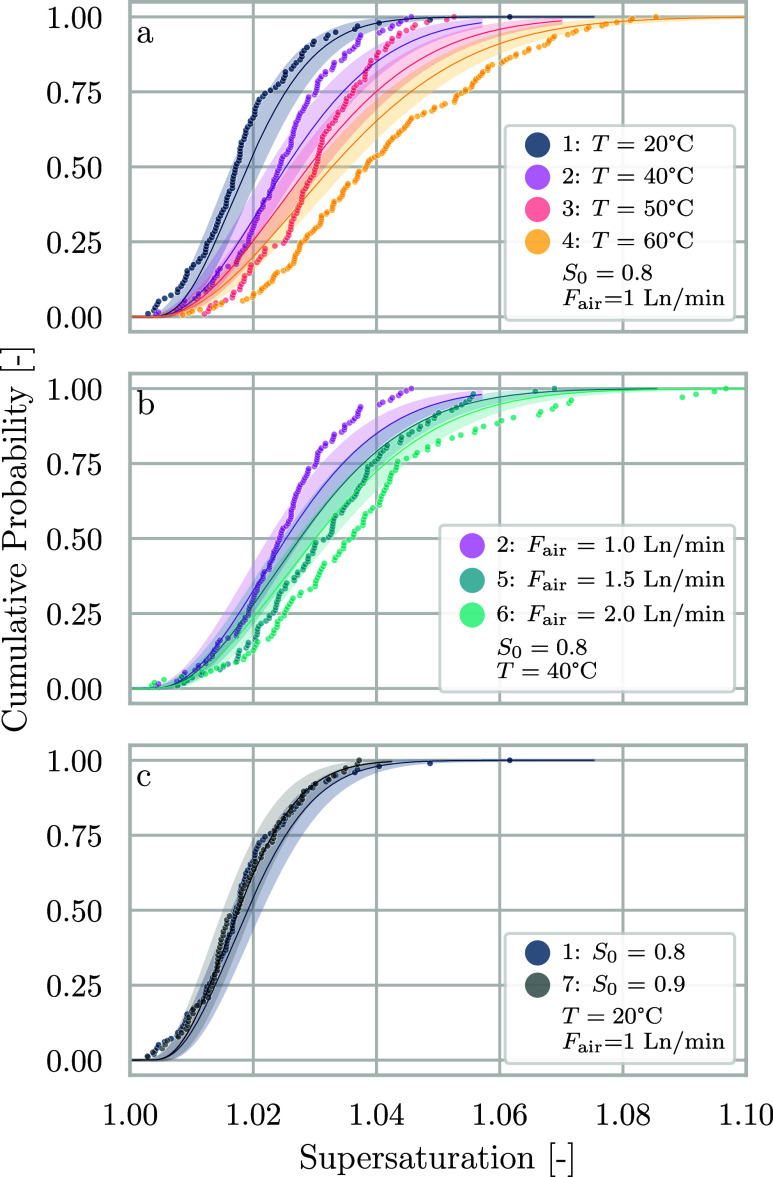
Cumulative probabilities of supersaturations at nucleation for NaCl at the conditions reported in Table 2. The scatter points represent experimental data, solid lines are model curves using fitted parameters, and shaded areas represent their 95% confidence intervals. (a) Series 1–4 at different levels of temperature. (b) Series 2, 5, and 6 at different flow rates of air. (c) Series 1 and 7 at different initial saturation ratios. Gas flow rates are measured in normal liters per minute, i.e., 0 °C and 1 bar.

Figure 4 shows the CDFs of supersaturation at nucleation under the same conditions. Supersaturation and dimensionless time τ essentially convey the same information, but supersaturation values can be compared with published data.

In the conversion from time to dimensionless time or supersaturation, time is inversely proportional to the evaporation rate *ṁ*_*v*_. This leads to an inversion of the CDF curves compared to those shown in Figure 3: as can be seen in Figure 4a and b, the conditions with the lowest evaporation rate are positioned to the left of the plots, corresponding to a smaller range of values of *S* at nucleation, while conditions at a high evaporation rate are on the right side. The validation experiment of series 7 shown in Figure 4c instead overlaps the reference series 1, as the conversion rescales the starting point (to *S* = 1) to show that the two conditions behave in the same way, as they are characterized by the same range of evaporation behavior.

### 3.3 Experimental Variability

**Figure 5:**
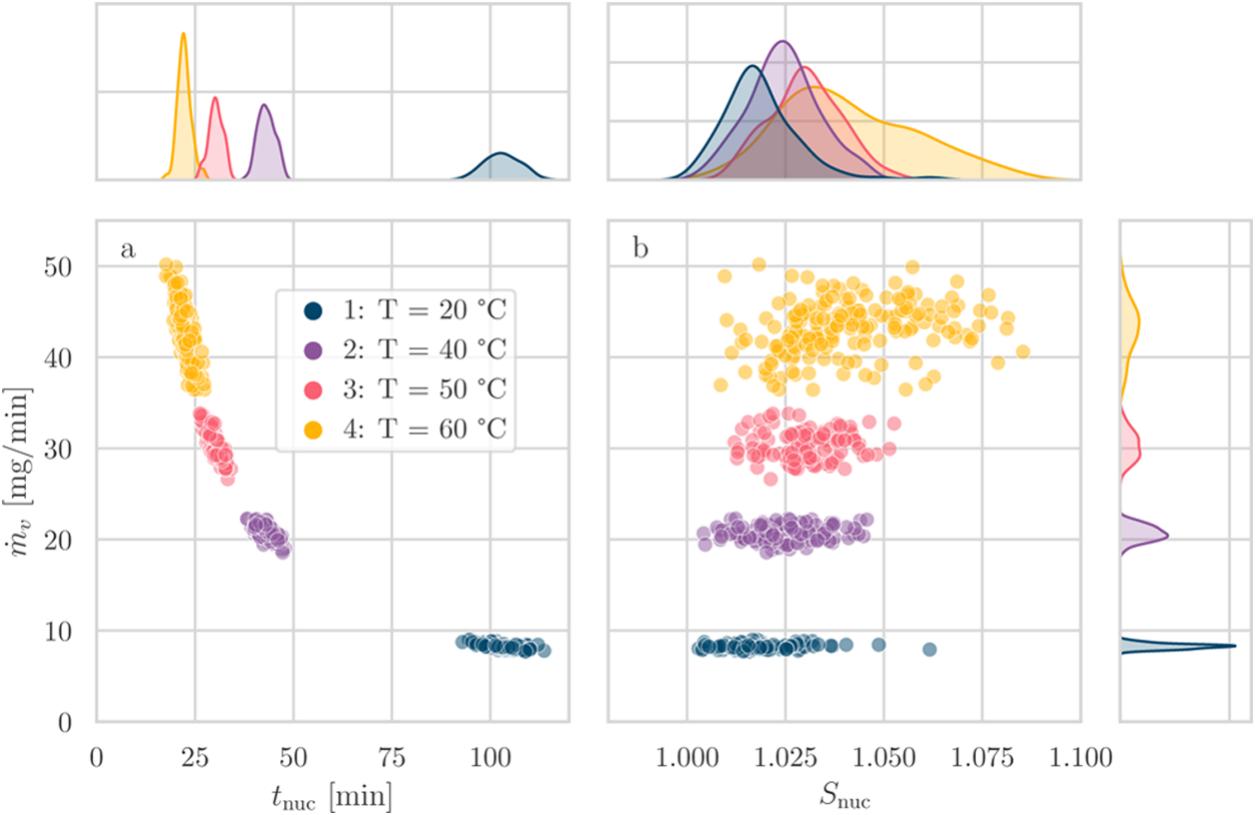
Marginal plots of nucleation time, supersaturation at nucleation, and evaporation rate for experiments at different temperatures at a fixed evaporation gas flow rate of 1 Ln/min and an initial saturation ratio of 0.8 (series 1–4).

**Figure 6:**
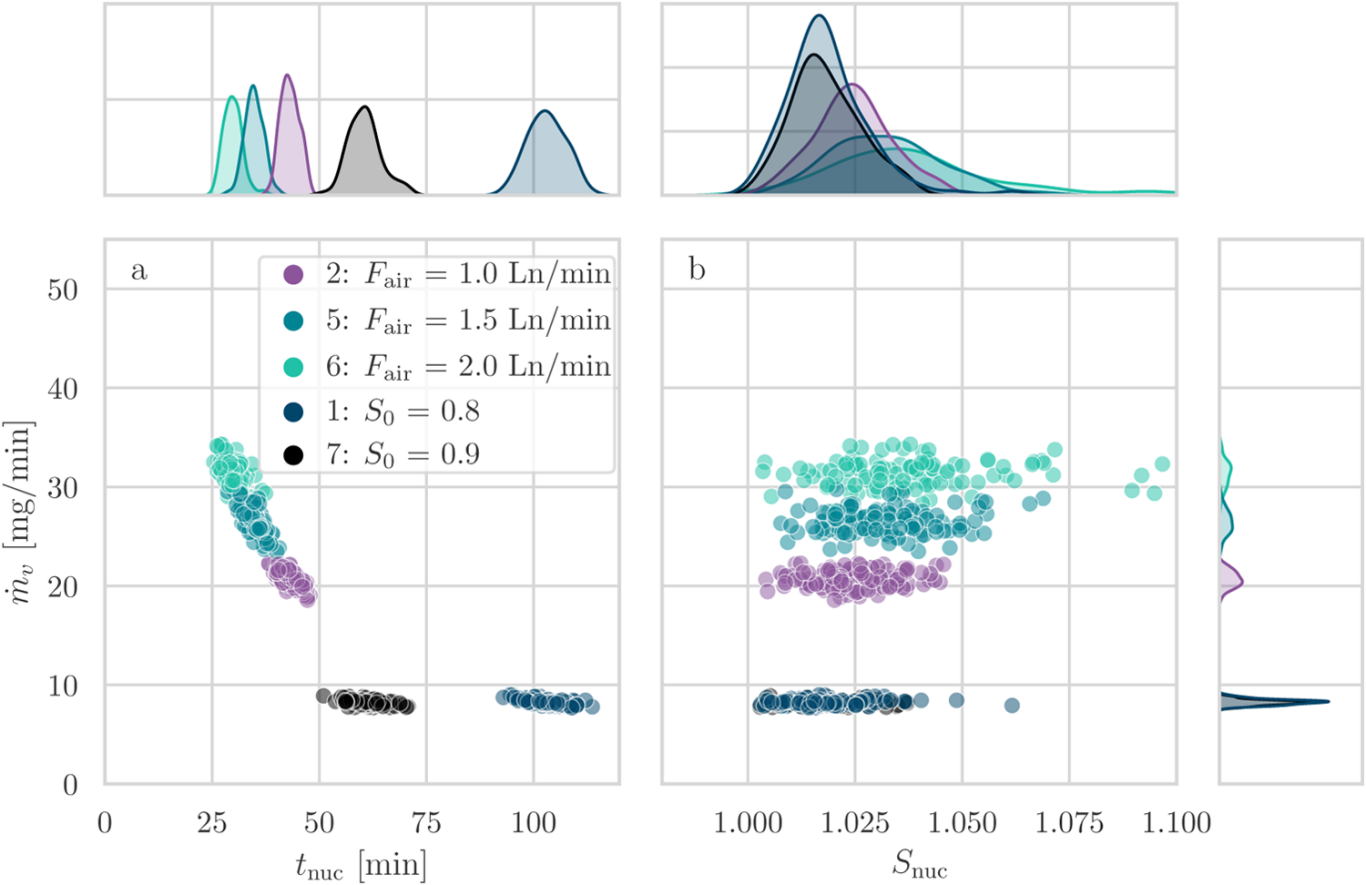
Marginal plots of nucleation time, supersaturation at nucleation, and evaporation rate for experiments at different evaporation gas flow rates at a fixed initial saturation ratio of 0.8 and a temperature of 20 °C (series 4–6) and different initial saturation ratios at a fixed evaporation gas flow rate of 1 Ln/min and a temperature of 40 °C (series 1 and 7).

Experimental variability originates from multiple sources, among which we can quantify (i) the evaporation rate *ṁ*_*v*_, (ii) the initial mass of solution *m*_0_, and (iii) the temperature *T*; this variability, together with the inherent stochasticity of primary nucleation, is reflected in the nucleation time *t*_nuc_ and the supersaturation level at nucleation *S*_nuc_. As we use the vial-specific, gravimetrically measured masses and evaporation rates associated with each nucleation time value in the parameter estimation, it is critical to describe and understand the variability of the measured quantities and their impact. We now focus on the observed variabilities in the evaporation rate, nucleation times, and supersaturation at nucleation since the vial-to-vial variabilities in the temperature and initial mass, as we show in Section 3.4, have a negligible impact on nucleation times and the supersaturation level at nucleation. In Figures 5 and 6, we show the marginal plots displaying the relationships between the first three quantities mentioned above, showing the four cases with varying temperatures (series 1–4) in the former and the remaining three experiments with related comparisons in the latter.

The marginal plots present two main panels with scatter plots, labeled (a) and (b), showing the relationship between the evaporation rate, which is the main driver of variability in evaporative crystallization, and the indicators of nucleation, i.e., *t*_nuc_ and *S*_nuc_. Above the two main plots, the marginal distributions of *t*_nuc_ and *S*_nuc_ are shown, while the marginal distribution of *ṁ*_*v*_ is plotted to the right.

It is worth highlighting that each point of the scatter plot corresponds to one experiment for which the evaporation rate is constant. We have shown this with dedicated experiments under the same conditions and with more frequent intermediate measurements in Figure S1 of the Supporting Information, where the loss of mass in each individual vial is shown to follow a constant evaporation rate.

Considering that the subset of experiments where the evaporation rate was varied using the temperature (Series 1–4), the marginal distributions of Figure 5 show that the nucleation times and evaporation rates are distinctly separated into clusters of data points, with an inverse relationship between nucleation times and evaporation rates. The width of the distributions of *ṁ*_*v*_ also provides useful information for the interpretation of the results: although experiments at a low temperature (*T* = 20 °C) exhibit the widest dispersion in nucleation times, their evaporation rates are narrowly distributed. While the evaporation rates of the collected experiments for an experimental series form a distribution, the evaporation rate can be considered constant for the single vial and run (S1 in the Supporting Information). On the other hand, when looking at the distribution of values of the supersaturation at nucleation in the marginal distribution and in Figure 5b, the ranges are found to be overlapping, as a consequence of the conversion from time to supersaturation. A rightward shift of the ranges of *S*_nuc_ is also present, with series 4 (highest temperature of *T* = 60 °C) exhibiting the widest distribution.

The horizontal dispersion of values for each cluster of points in Figure 5a represents the overall observed variability of the set of experiments. Part of this is caused by the variability in the evaporation rate: in fact, these conditions show a clear trend of increasing nucleation time with decreasing evaporation rates. The scatter plot of *ṁ*_*v*_ as a function of *S*_nuc_ (Figure 5b) better represents the stochasticity inherent to primary nucleation when considering points at a fixed value of the evaporation rate.

When we consider the variability of nucleation indicators (time and supersaturation at nucleation), we observe that it is larger at low temperatures when considering time and at high temperatures when considering supersaturation. This behavior is explained by the range of values of the evaporation rate and their variability: the evaporation rate appears in the conversion from time to supersaturation, where the supersaturation and the evaporation rate are inversely proportional (see S3 in the Supporting Information).

Next, we consider the marginal plot for the remaining experimental conditions where we vary either the evaporation rate through the flow rate of air or the initial saturation ratio in Figure 6. The same considerations on the evaporation rate made for Figure 5 apply.

The same considerations can be made regarding the stochasticity of primary nucleation and experimental variability when considering the clusters of series 4–6: in Figure 6a, the variability in the nucleation times is very similar at different levels of the flow rate of air, as the temperature is fixed at 40 °C, while Figure 6b shows that increasing values of the evaporation rate lead to a larger variability in *S*_nuc_. A comparison of series 1 and 7 shows the repeatability of our method despite a difference in initial concentrations: the ranges of evaporation rates and supersaturation levels at nucleation overlap, while the nucleation times are delayed in the case of the low-concentration solution.

### 3.4 Analysis of Variability

We assessed the impact of the measured quantities *m*_0_, *ṁ*_*v*_, and *T* on the nucleation metrics (nucleation times and supersaturation levels) following the procedure described in Section 2.7. For the sake of simplicity but without loss of generality, we focused the analysis on the conditions and data belonging to series 1 at *T* = 20 °C, *S*_0_ = 0.8, and a flow rate of air of 1 Ln/min. As previously mentioned, we estimated the fraction of variance of nucleation times, dimensionless times, and supersaturation levels attributable to the three quantities *m*_0_, *ṁ*_*v*_, and *T* and calculated the residual fraction not explained by them, which we can ascribe to the stochasticity of primary nucleation primarily and other factors not accounted for by adjustments to the individual parameters secondarily.

For *t*_nuc_, a substantial fraction of the total variance is linked to the variability in *ṁ*_*v*_ (>45%). This means that better control of *ṁ*_*v*_ would significantly reduce the observed variability in nucleation times. The other contributions, originating from the variability in the initial mass and the fluctuation in temperature, account for less than 0.5% of the variance and do not significantly influence the variance of nucleation times.

In contrast, for τ_nuc_ and *S*_nuc_, the majority of the variance (>96%) remains unexplained by changes in *m*_0_, *ṁ*_*v*_, or *T*. This is because both quantities are calculated in such a way that removes the influence of variability of the evaluated parameters. Their distribution hence can be attributed to the inherent stochasticity of nucleation.

Overall, these results highlight the impact of evaporation rate on the directly observable nucleation time.

### 3.5 Parameter Estimation and Nucleation Rate

Next, we performed parameter estimation with the procedure outlined in Section 2.5 on the data for series 1–4. The parameters obtained in this way are reported in Table 3.

**Table 3:** Fitted Parameters Using the CNT Expression of the Nucleation Rate and Series 1–4, with 95% Confidence Intervals Obtained by the Bootstrap Method Using 1000 Samples

parameter	value	confidence interval	unit
*A*	9.4·10^10^	[8.57·10^10^, 1.07·10^11^]	[m^–5^]
*B*	1201	[549, 2077]	[K^3^]

The model predictions are shown in Figure 3 as solid lines, with the 95% confidence intervals represented as a shaded area. The remaining data sets (series 5–7) were not used for parameter estimation, but the model can still describe their nucleation times reasonably well. The comparison of series 4 with series 5–6 in Figure 3b shows that the model can describe the effect of evaporation rate through the evaporation model even for conditions not used for parameter estimation. Finally, in Figure 3c, we compare series 1 to a set of data using a different initial concentration of the solution, corresponding to *S*_0_ = 0.9 (series 7). As this condition is essentially a repetition of series 1 with a higher initial concentration, we used it as validation of the experimental reproducibility of our procedure and the adaptability of the model to different starting conditions. The deviations at short times in Figure 3 may arise due to the scarcity of data points in that region, differences in heterogeneous sites in different vials, violations of the single-nucleus hypothesis, or other factors not accounted for by the model. The tails of the CDFs (both short and long times) may be less populated and are subject to higher statistical uncertainty. These factors could lead to minor deviations between the model simulations and the experimental results. Furthermore, the parameters are estimated using the dimensionless time τ, which correlates to the saturation ratio *S* better than the time *t* as it removes the variability linked to the evaporation rate. Additionally, Figure 3 shows all experimental series on the same axis, where series 2–4 are compressed on the horizontal axis compared to the case of series 1, making its fit appear less aligned with the experimental distribution.

**Figure 7:**
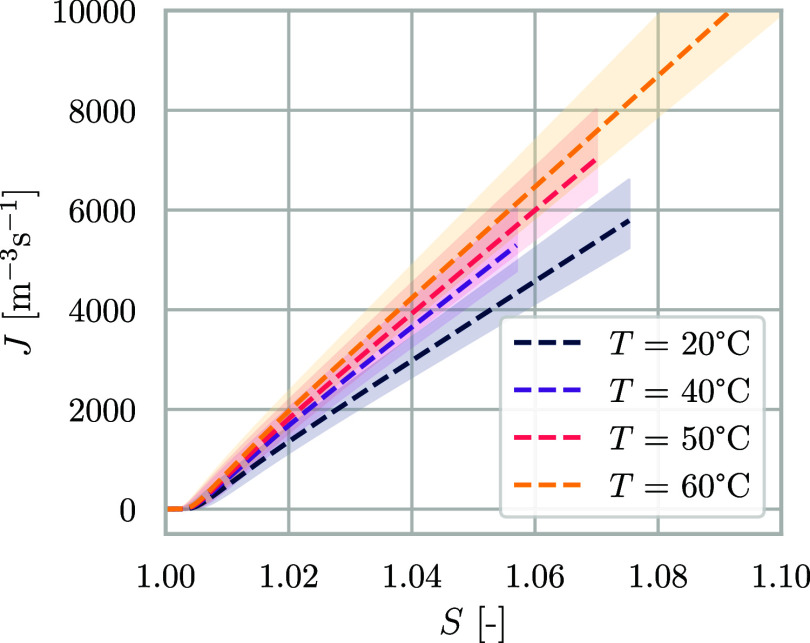
Calculated nucleation rates as a function of supersaturation for model experiments based on averages of series 1–4. Error bands are calculated using the 95% confidence intervals on the nucleation rate parameters.

The nucleation rate expression used in this work (eq 11) is a function of supersaturation and temperature. In Figure 7, we show plots of *J* as a function of *S* for simulations at different temperatures, representing the average experiment of series 1–4, with the shaded area representing the 95% confidence interval on parameters *A* and *B*. The temperature dependence of the nucleation rate is weak, with supersaturation remaining the main factor influencing the value of *J*. For the case of NaCl, where solubility is almost temperature-insensitive, temperature mainly plays a role in the pre-exponential factor of the nucleation rate through diffusivity, where it promotes the frequency of collisions, leading to the formation of critical nuclei. Other model systems with temperature-sensitive solubility would be negatively impacted by high temperatures. Sodium chloride presents a small metastable zone width, as shown by the region in Figure 7, in which the nucleation rate is zero. This means that nucleation occurs at small values of supersaturation and that the entity of the energy barrier at the experimental conditions of our work is also small. This does not, however, prevent the manifestation of the stochastic nature of nucleation, i.e., the presence of inherent variability, which can be seen when considering supersaturation at nucleation, as already shown in Figure 4.

## 4 Discussion

Sodium chloride crystallization kinetics are widely studied due to their importance as a model system for understanding nucleation phenomena. Most available data derive from molecular dynamics (MD) simulations or small-scale experiments, as summarized by Finney and Salvalaglio.^18^ While MD simulations predict high supersaturation values (2 < *S* < 5) in extremely small simulated volumes and microscale experiments report nucleation events at 1.1 <*S* < 2.2,^23−25,29,33,45,62−65^ these approaches differ from crystallization in larger systems.

Our experimental results show that NaCl nucleation at a larger scale occurs at consistently lower supersaturation levels (*S* < 1.04) compared to the values seen at the microdroplet scale, as seen in Figures 5 and 6. This behavior of nucleation occurring at lower values of *S* in larger vessels is predicted by the expression of nucleation probability (eq 10) and is in line with other experimental observations reported in the literature.^26,66^ Nucleation rates, shown in Figure 7 for series 1–4, are in comparable range with those measured by Flannigan et al.^26^ (*J* < 1000 m^–3^s^–1^ in the range *S*_nuc_ = [1,1.02]), considering that the literature studies obtained single values from isothermal induction time measurements, while our experimental conditions present time-dependent volume, supersaturation, and thus nucleation rate, and their measurements are performed at a cooling rate of 5 °C min^–1^, affecting the metastable zone width.

The use of only a fraction of our data set to estimate parameters (series 1–4) shows that our model well describes the effect of the evaporation rate, even when it is determined by the flow rate of air instead of the temperature level. Estimating the parameters using only data at a constant flow rate of evaporation gas where the evaporation rate was determined by different levels of temperature extends reasonably to describe conditions where the change in evaporation rate comes directly from the flow rate of evaporation gas at a fixed temperature. The observed behavior of nucleation with the evaporation rate is consistent with the analogous method of inducing supersaturation by cooling: as the rate of change in supersaturation increases, samples nucleate deeper into the metastable zone. Changing the initial concentration of the solution does not affect the model’s capability to describe the experimental data either; this confirms the reproducibility of the experimental procedure.

The close agreement between the experimental data and the best fit of the model supports the notion that the stochastic model used here describes the phenomenon of NaCl nucleation in vials with reasonable accuracy. We acknowledge, however, that it has not been possible for us to directly validate the key underlying assumption of the stochastic model, i.e., that only a single primary nucleus forms per vial. In fact, the observation of very small metastable zones such as those that we see here may alternatively be interpreted as a process where nucleation is very fast and multiple primary nuclei form. In such a case, the observed distribution in nucleation times would be due to an experimental error.

For this reason, we carefully assessed all relevant sources of experimental error through a variance analysis, which revealed that experimental errors do not have a relevant effect on the distribution of dimensionless nucleation times and supersaturation at nucleation. This provides indirect evidence that the single-nucleus assumption applies to the experiments studied here.

Finally, let us reflect in more general terms on the use of evaporation experiments to estimate nucleation parameters. In contrast to induction time measurements at constant supersaturation, the increase in solute concentration over time during evaporation experiments ensures that nucleation takes place in all vials within a reasonable period of time. The evaporation method is not affected by premature nucleation that arises in induction time experiments where the desired supersaturation level is generated by crash-cooling. In these two aspects, evaporation performs similarly to cooling experiments, where metastable zone widths are used to infer nucleation kinetics. The isothermal nature of evaporation experiments, however, is beneficial as it allows for the use of simpler, temperature-independent nucleation rate expressions. Given that the supersaturation is generated as a result of a reduction in volume instead of a temperature change, the method can be used for arbitrary compounds, independent of how their solubility depends on temperature. For these reasons, the evaporation method presented here promises to be of use for a wide range of nucleation measurements in the future.

The problem of unnucleated samples is reduced partly by the polythermal approach to cooling crystallization, where the limit is set on the lowest temperature achieved rather than on the longest experiment duration. Choosing a technique where the driving force for nucleation changes in time, be it by changing either the temperature or the mass of solvent, can ensure that nucleation events will eventually take place in a reasonable amount of time.^67,68^

## 5 Conclusions

In this study, we introduced a novel approach for measuring nucleation rates using evaporative crystallization in commercially available instrumentation at the milliliter scale. This method was applied to sodium chloride in water, a system where traditional cooling crystallization methods are impractical due to the negligible temperature dependence of solubility. We combined experimental measurements with a first-principles nucleation model, which accounts explicitly for a temperature-dependent nucleation rate. We demonstrated the reproducibility of this method through experiments at different evaporation rates (achieved using different flow rates of air) and different initial concentrations.

The observed supersaturation at nucleation and the calculated nucleation rates align well with the values reported in the literature,^26,66^ which underscores the reliability of evaporative crystallization for measuring nucleation rates. While evaporative crystallization is more labor-intensive than cooling crystallization, it enables a wider range of experimental conditions to be explored, particularly for systems where solubility is temperature-insensitive or systems that are prone to thermal degradation.

Estimating nucleation rates from evaporation experiments in stirred vials is suitable for the case of compounds exhibiting temperature-insensitive solubility and contributes to enhancing our understanding of the underlying phenomena in the crystallization process, as vials incorporate the use of stirring and the presence of multiple interfaces that may promote nucleation (i.e., glass, air, impeller, dust particles).

## Supporting Information Available

Characterization of the evaporation rate, experimental variability and outlier rejection, characterization of the actual vial temperatures, conversion from time to supersaturation, and sensitivity analysis (PDF)
