# Supplementary material for: Developing a Vial-Scale Methodology for the Measurement of Nucleation Kinetics Using Evaporative Crystallization: A Case Study with Sodium Chloride
Source: Cryst Growth Des. 2025 Apr 4;25(8):2498–509. doi: 10.1021/acs.cgd.4c01722 (PMC12006966; doi:10.1021/acs.cgd.4c01722)
Supplement: Supplementary file 1 — cg4c01722_si_001.pdf [file cg4c01722_si_001.pdf]

Supporting Information: Developing a vial-scale methodology for the measurement of nucleation kinetics using evaporative crystallization: a case study with sodium chloride

Michele Chen, Leif-Thore Deck,[a] Luca Bosetti, and Marco Mazzotti*

Institute of Energy and Process Engineering, ETH Zürich, 8092 Zürich, Switzerland

E-mail: marco.mazzotti@ipe.mavt.ethz.ch

[a]Current affiliation: Yusuf Hamied Department of Chemistry, University of Cambridge, Cambridge CB2 1EW, United Kingdom

S1 Characterization of the evaporation rate

The assumption of constant evaporation rate[S1] was tested by frequent gravimetric measurements of an undersaturated aqueous NaCl solution with initial saturation ratio of 0.8. Measurements were taken from vials placed under isothermal conditions ($T = 40\,°\mathrm{C}$) in the Crystalline, stopping the gas flow (1 Ln/min) every 15 minutes for measurements and resuming, for all vials at the same time. Evaporation rates were calculated for the sub-intervals and over the entire evaporation.

As shown in Figure S1, the evolution of the solution mass in different vials was evaluated over the typical time needed for nucleation to occur. The loss of mass due to the evaporation was modeled using equation S1, where the fitted parameters confirmed the constant nature of the evaporation rate.

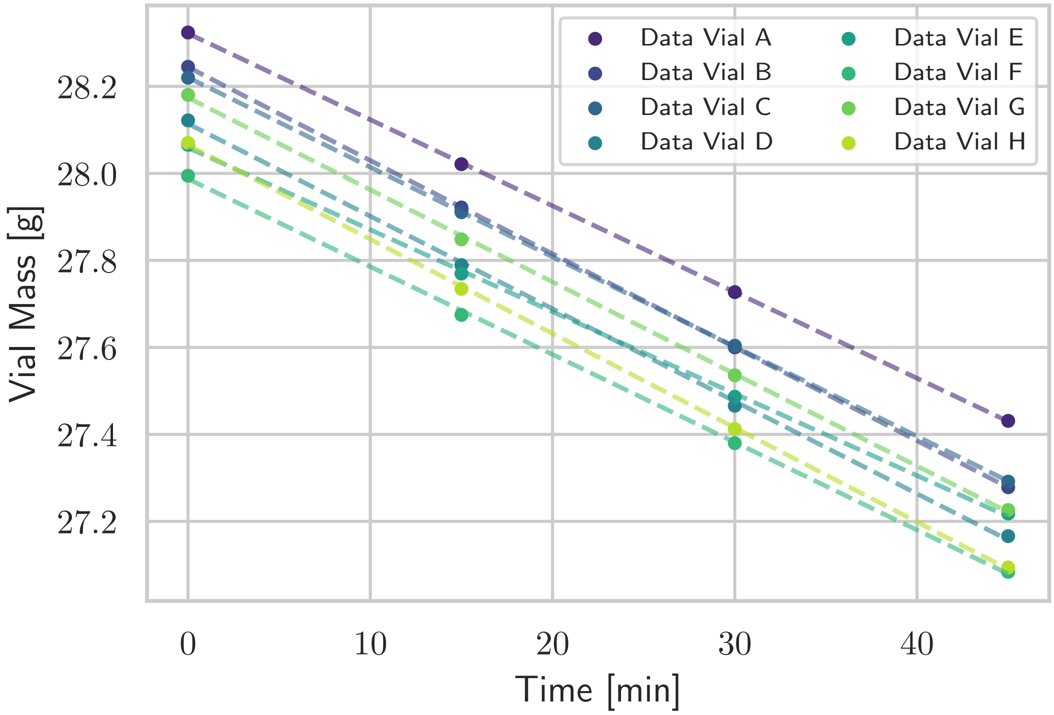

13 Figure S1: Evolution of the solution mass due to evaporation at 40 °C and 1 Ln/min. Solid
14 points represent the experimentally measured masses, while dotted lines represent the model
15 fit.

$$m_{solution}(t) = m_0 - at \tag{S1}$$

16 Using a linear expression to express the evaporation rate, we obtain a distribution of
17 evaporation rates, of which the mean value is $\dot{m}_{v,mean} = 0.0206\,\mathrm{g\,min^{-1}}$, with a standard
18 deviation of $\sigma_{\dot{m}_v} = 0.0009\,\mathrm{g\,min^{-1}}$. For each of these fits, we determined the R^2. From
19 the distribution of R^2 values,we obtain that $R^2_{\mathrm{mean}} = 0.9998$ with standard deviation $\sigma_{R^2} =$
20 0.0002.

21 Measurements were performed also at $T = 20\,°\mathrm{C}$, showing the same result.

S2 Experimental variability and Outlier Rejection

Variability of the initial mass of solution $m_0 = m_{\mathrm{NaCl},0} + m_{\mathrm{H_2O},0}$ can be evaluated using pair plots and compared to the indicators of nucleation t_{nuc} and S_{nuc}.

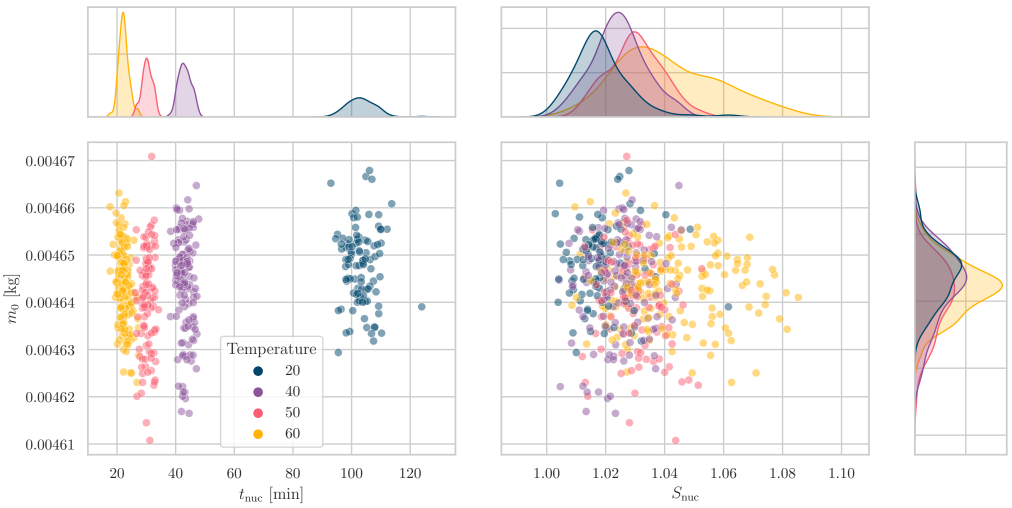

Figure S2: Marginal plots of nucleation time, supersaturation at nucleation and initial solution mass for experiments at different temperature, at fixed evaporation gas flow rate of 1 Ln/min and initial supersaturation 0.8.

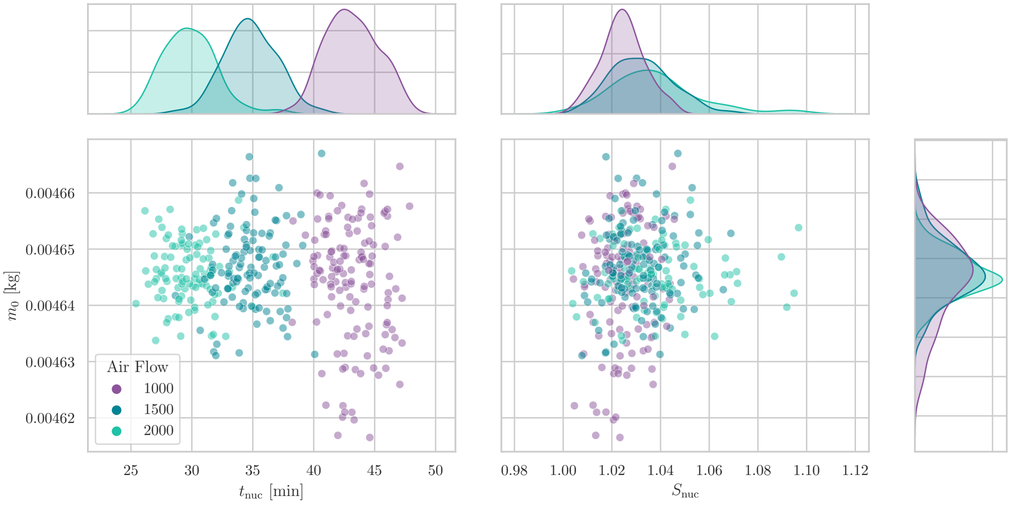

28 Figure S3: Marginal plots of nucleation time, supersaturation at nucleation and initial solution mass for experiments at different evaporation gas flow rates, at fixed initial supersaturation of 0.8 and temperature of 20°C.

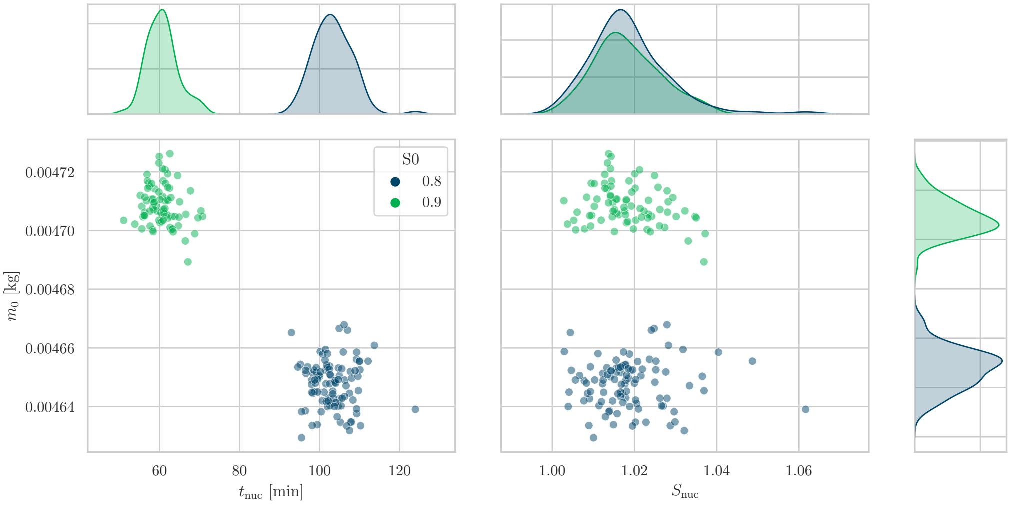

31 Figure S4: Marginal plots of nucleation time, supersaturation at nucleation and initial solution mass for experiments at different initial supersaturation, at fixed evaporation gas flow rate of 1 Ln/min and temperature of 20°C.

S2.1 Characterization of the Actual Vial Temperatures

Average temperatures were used instead of nominal set point values in the parameter estimation and calculation of nucleation rates. As shown in Table S1, the deviation is larger as the temperature increases.

Table S1: Average measured temperatures in the vials using thermocouples connected to a PicoLog TC-08 temperature logger.

Vial ID	A	B	C	D	E	F	G	H
T_{SP} [°C]				T [°C]				
20	23.47	21.29	20.23	20.1	20.14	19.88	22.02	22.61
40	33.33	33.23	33.17	33.04	33.65	33.93	33.26	33.55
50	37.89	38.65	38.33	39.58	39.42	38.5	38.91	36.36
60	42.8	43.01	44.02	47.08	44.75	47.26	42.96	43.01

S3 Conversion from Time to Supersaturation

When plotting the CDF of nucleation times t_{nuc} and that of supersaturations at nucleation S_{nuc}, we notice that the distributions present a stretching effect. This can be explained by making explicit the relationship between S and t:

$$S = \frac{C(t)}{C^*_{\mathrm{NaCl}}} = \tag{S2}$$

$$= \frac{m_{\mathrm{NaCl}}}{C^*_{\mathrm{NaCl}}} \frac{1}{V(t)} = \tag{S3}$$

$$= \frac{m_{\mathrm{NaCl}}}{C^*_{\mathrm{NaCl}}} \frac{\rho(x_{\mathrm{NaCl}}, T)}{m_{\mathrm{NaCl}} + m_{\mathrm{H_2O}}(t)} \tag{S4}$$

If we consider an empirical correlation for $\rho(x_{\mathrm{NaCl}}, T) = a + bx_{\mathrm{NaCl}} + cT^2$ and express the time-dependent mass of water with Equation 1 and the mass fraction of NaCl with Equation 2, we can continue by regrouping some terms:

$$A = \frac{m_{\mathrm{NaCl}}}{C^*_{\mathrm{NaCl}}} \tag{S5}$$

$$B = a + cT^2 \tag{S6}$$

$$C = bm_{\mathrm{NaCl}} \tag{S7}$$

$$D = m_{\mathrm{NaCl}} + m_{\mathrm{H_2O},0} \tag{S8}$$

The final equation becomes:

$$S = \frac{AB}{D - \dot{m}_v t} + \frac{AC}{(D - \dot{m}_v t)^2} \tag{S9}$$

The conversion from t to S is thus nonlinear, resulting in some stretching of the values when performed. The stretching is also temperature dependent, due to the contribution of the density.

S4 Sensitivity Analysis

S4.1 Error on solubility

To analyze the solubility of NaCl in water as a function of temperature, a linear correlation was developed using data from Flannigan et al.[S2]. The performance of this correlation was evaluated against both the Flannigan dataset and a broader experimental dataset aggregated from multiple sources.

The selected data were fitted using a linear model implemented with the LinearRegression function in SciKit Learn, yielding the correlation:

$$C^*_{\mathrm{NaCl}}(T) = 0.3541 + 0.0002\,T \tag{S10}$$

The percent error for each data point was calculated using the formula:

$$\text{Percent Error} = \frac{\text{Experimental-Predicted}}{\text{Experimental}} \cdot 100 \tag{S11}$$

This analysis was performed for both the Flannigan dataset and the entire experimental dataset to evaluate model accuracy. For the Flannigan dataset, the average percent error was found to be 0.254%, whereas for the entire dataset, it was 0.490%. The model fit is visualized in Figure S5, which shows the experimental data points (color-coded by source) alongside the linear model. The percent error for individual points is also annotated in the figure.

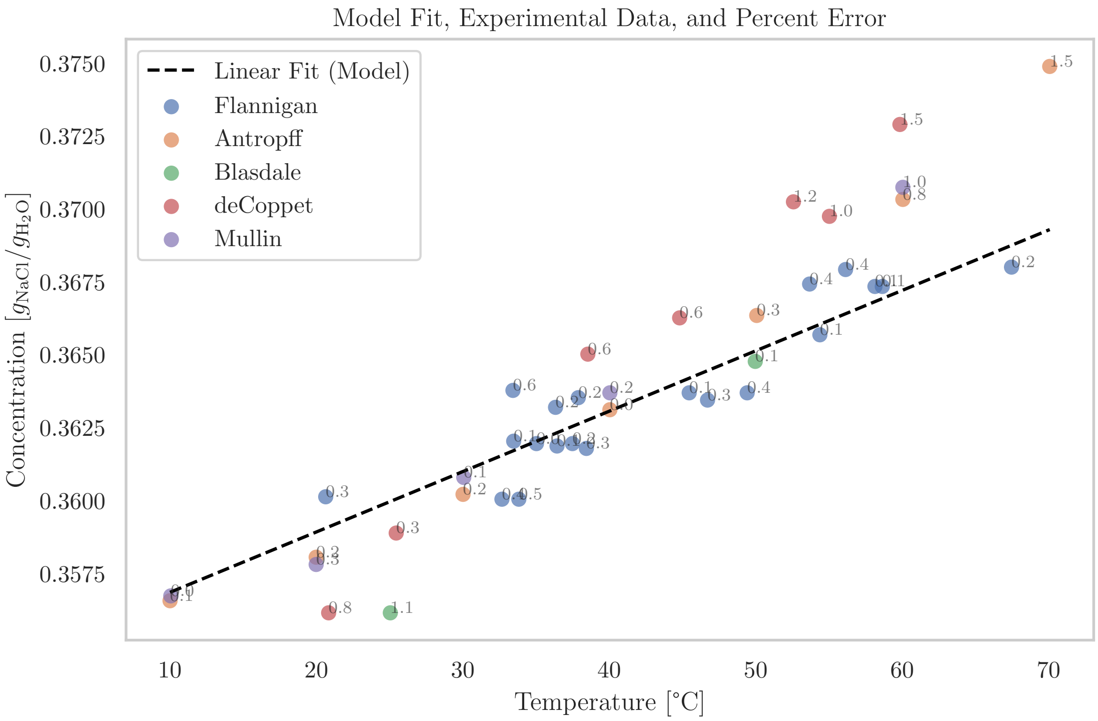

Figure S5: Linear correlation of NaCl solubility in water as a function of temperature. The dashed black line represents the linear model obtained using data from Flannigan et al.[S2] ($C^*_{NaCl}(T) = 0.3541 + 0.0002\,T$). Experimental data points are color-coded by source, with percent error between experimental values and model predictions annotated for each data point.

S4.2 Error on gravimetric measurements

All mass measurements were performed using a Mettler Toledo AX205 analytical balance. According to the manufacturer's specifications, this balance provides mass measurements with an uncertainty on the order of ±0.2 mg over the mass range of interest. For initial sample masses on the order of 20–30 g, this corresponds to a relative uncertainty of approximately 0.001%. Specifically, for a 25 g sample, an absolute uncertainty of ±0.2 mg translates to a relative uncertainty of about 0.0008%. This uncertainty closely reflects what we observed experimentally.

Each mass measurement involves an uncertainty of Δm (e.g., ±0.2 mg). Assuming the uncertainties in repeated mass measurements are independent and similar in magnitude, the

uncertainty in the mass difference (m_0–m_1) is given by:

$$\Delta(\Delta m) = \sqrt{(\Delta m_0)^2 + (\Delta m_1)^2} \tag{S12}$$

For equal uncertainties Δm in each measurement, this simplifies to:

$$\Delta(\Delta m) = \Delta m\sqrt{2} \tag{S13}$$

Thus, if each measurement is uncertain by ± 0.2 mg, the difference has an uncertainty of approximately ± 0.283 mg. When calculating the evaporation rate, this absolute uncertainty in mass difference is divided by the measured time, assuming the time measurement is exact or its uncertainty is negligible. The resulting uncertainty in $\dot{m}_v$ depends on the magnitude of the mass difference: larger mass differences relative to the 0.2 mg scale uncertainty produce smaller percentage errors, while smaller mass differences yield higher relative errors.

The supersaturation, S, is computed using Equation 4. Uncertainties in $m_{\mathrm{H_2O},0}$ and $\dot{m}_v$ propagate to $m_{\mathrm{H_2O}}(t)$. The combined uncertainty is calculated using standard propagation of errors. For uncorrelated variables, uncertainties add in quadrature:

$$\Delta m_{\mathrm{H_2O}}(t) = \sqrt{(\Delta m_{\mathrm{H_2O},0})^2 + (t^2(\Delta \dot{m}_v)^2)} \tag{S14}$$

The solubility of NaCl at a given temperature T is taken from a known correlation, which we have evaluated in Subsection S4.1.

The supersaturation $S(t)$ is calculated using Equation 4, its uncertainty primarily arises from the uncertainties in m_{NaCl}, $m_{\mathrm{H_2O}}(t)$, ρ, and ultimately $V(t)$.

To handle the complexity of multiple interdependent variables, we employed a Monte Carlo method. We define probability distributions for the uncertain input parameters (e.g., normal distributions centered on measured values with standard deviations equal to the identified uncertainties) and generate a large number of random realizations of these para-

meters. For each realization, we recalculate the evaporation rate, solution composition, density, volume, and supersaturation. By examining the distribution of the resulting supersaturation values, we obtain a statistical estimate of the mean and standard deviation of S. For the experimental conditions reported in Table 2, we obtain a relative uncertainty in S between 0.07% and 0.27%, small enough not to affect our determination of nucleation.

References

(S1) Ramos Ojeda, N. A.; Kind, M. Transferring Crystallization Conditions from Small to Larger Scale for Achieving Targeted Crystal Morphologies of an Active Pharmaceutical Ingredient. *Crystals* **2024**, *14*.

(S2) Flannigan, J. M.; MacIver, D.; Jolliffe, H.; Haw, M. D.; Sefcik, J. Nucleation and Growth Kinetics of Sodium Chloride Crystallization from Water and Deuterium Oxide. *Crystals* **2023**, *13*, 1–28.
